# Supplementary material for: Dynamin-Related Proteins Enhance Tomato Immunity by Mediating Pattern Recognition Receptor Trafficking
Source: Membranes (Basel). 2022 Aug 1;12(8):760. doi: 10.3390/membranes12080760 (PMC9415932; doi:10.3390/membranes12080760)
Supplement: Supplementary file 1 [file membranes-12-00760-s001.zip › Supplementary Materials.pdf]

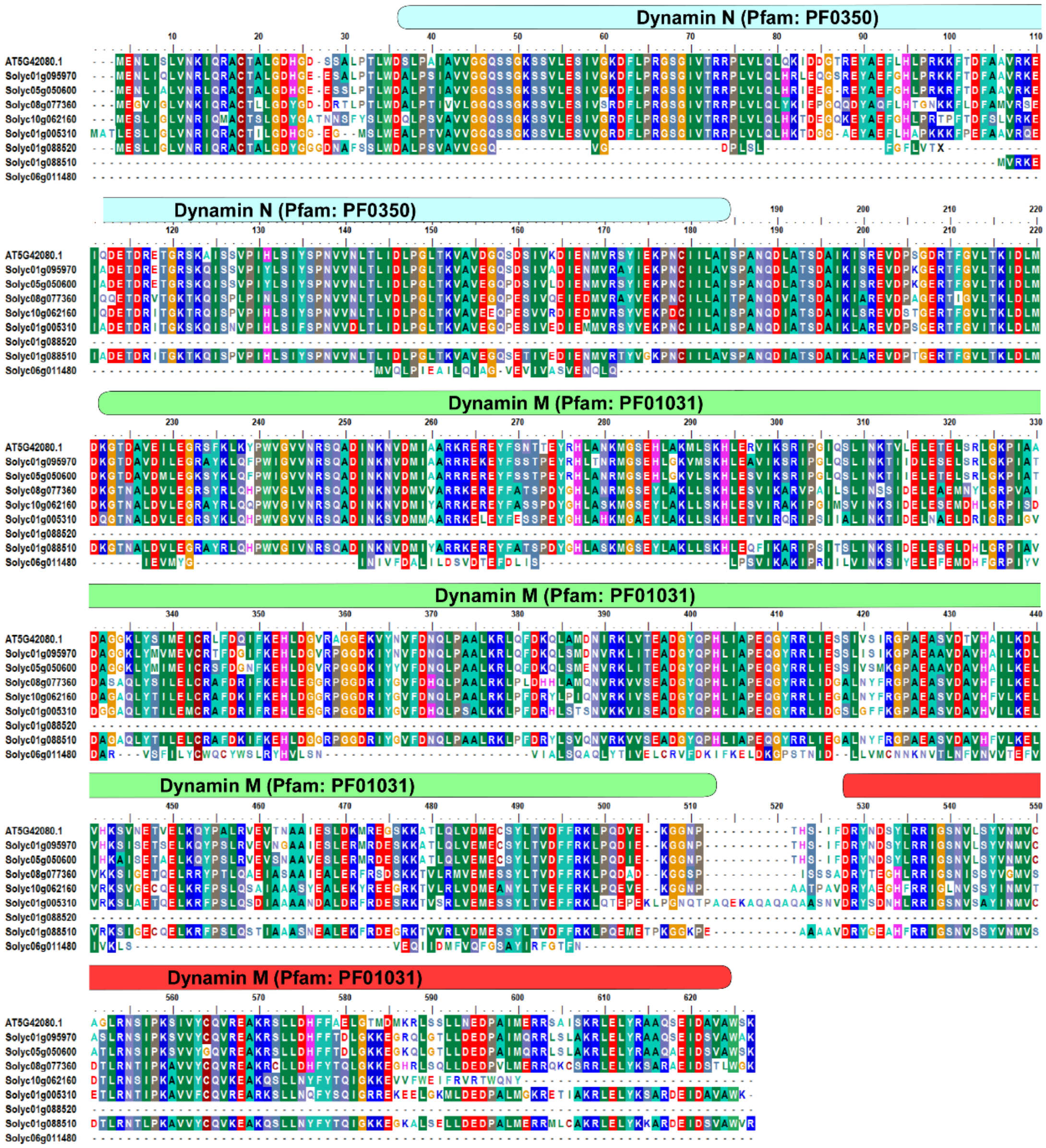

Figure S1. Alignment of tomato DRP1 orthologues.

ClustalW Alingment of *A. thaliana* DRP1A and tomato DRP1 orthologues: Solyc01g095970, Solyc05g050600, Solyc01g005310, Solyc08g077360, Solyc10g062160, Solyc01g088510, Solyc01g088520 and Solyc06g011480. Protein domain analysis was made using the HMMER database and shown in the scheme.

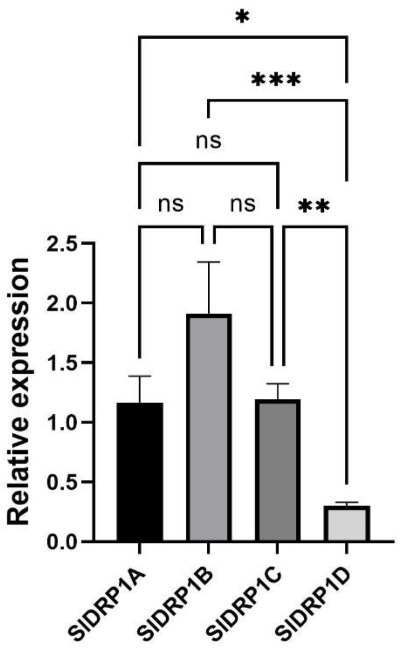

Figure S2. SlDRP1s expression in tomato leaves.

M82 tomato RNA was used for measuring expression level of SlDRP1s: SlDRP1A (Solyc01g095970), SlDRP1B (Solyc05g050600), SlDRP1C (Solyc01g005310) and SlDRP1D (Solyc08g077360). Relative expression was determined comparing to RLP8 housekeeping gene ($2^{Ct\ Hkp - Ct\ SlDRP1X}$). Three RNA biological replicates were used, each reaction was performed in triplicate. Media and SD is presented. Statistical analysis used was One-way ANOVA and post-test ad-hoc Kluskal-Wallis. * p-value ≤ 0.05, ** p-value ≤ 0.01, *** p-value ≤ 0.001.

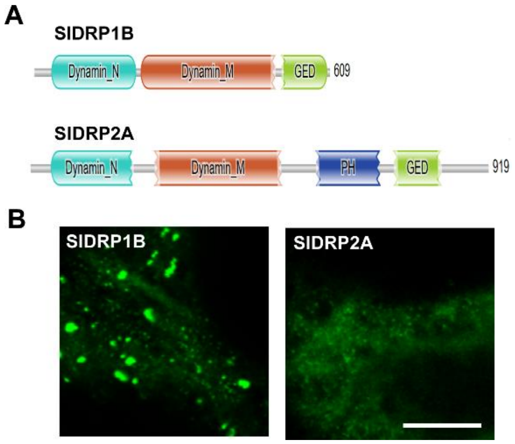

Figure S3. SlDRP1B and SlDRP2A comparison

A) Scheme of protein domains present in SlDRP1B and SlDRP2A (HMMER database). **B)** Subcellular distribution of SlDRP1B and SlDRP2A was analyzed through confocal microscopy of *N. benthamiana* epidermal cells transiently expressing SlDRP1B-GFP and SlDRP2A-GFP. Images were acquired using a Zeiss LSM 780 Confocal Microscope. Squares indicate the inset region. Scale bar 10 µm.

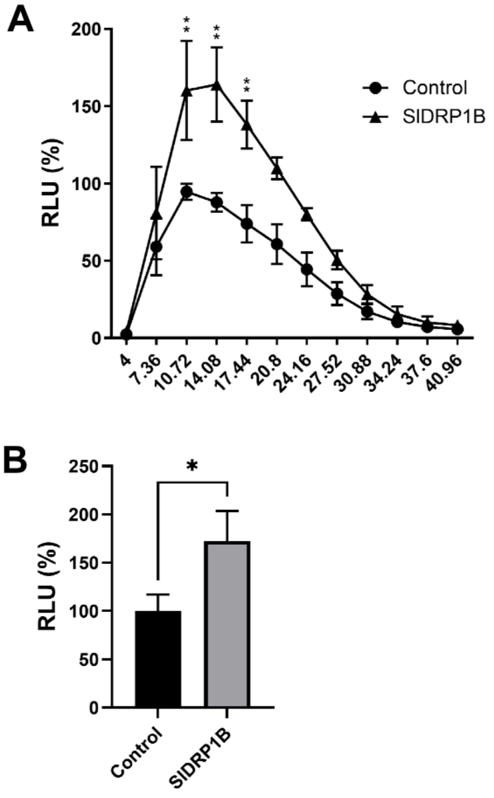

Figure S4. Effect of overexpression of SlDRP1B on FLS2-mediated oxidative burst. Leaf disks of *N. tabacum* transiently expressing SlDRP1B-tagged or free-tag (control) were harvested 48 h after transformation. Luminescence (RLU) was measured immediately after flg-22 application. Error bars represent the average +SEM of 5 independent experiments, N=12. (**A)** The average value of the control peak was defined as 100%. Time points represent the average ± SEM. Asterisks indicate significant differences with control treatment (Two-way ANOVA, *P < 0.05, **P<0.01). (**B)** Total RLU was calculated and normalized to control. One-way ANOVA, P < 0.05.

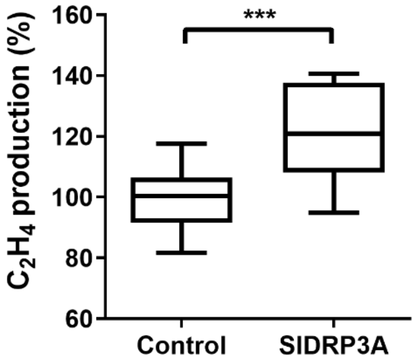

Figure S5. Effect of overexpression of SlDRP3A on LeEIX2-mediated defense. Leaf disks of *N. tabacum* transiently expressing SlDRP3A-tagged or free-tag (control) were harvested 48 h after transformation. Ethylene biosynthesis was measured four h after EIX application. The average value of control was defined as 100%. Boxplots represent minimum to maximum values, with boxes representing the inner-quartile ranges, whiskers representing the outer quartile ranges, and the line in the box representing the median, of 3 independent experiments, with asterisks denoting significant differences to control treatment (t-test, ***P<0.001).

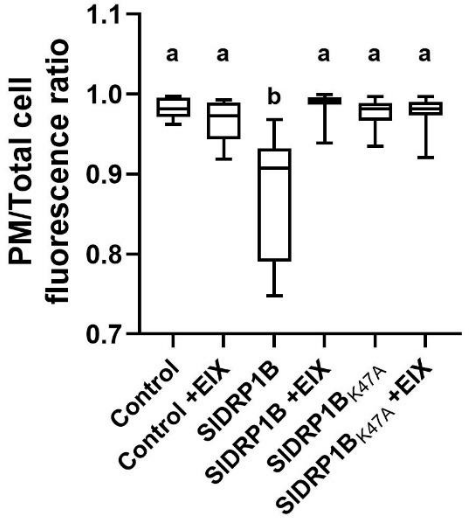

Figure S6: LeEIX2 localization ratio at PM.

N. benthamiana leaves transiently expressing LeEIX2-GFP and free mCherry (Control), SlDRP1B-mCherry and SlDRP1BK47A were treated with EIX (1 µg mL−1 tissue) or water at the petiole 40 h after transformation and visualized after 15 min. LeEIX2 fluorescence ratio at the PM/total cell fluorescence was calculated using the data set from Figure 5 (Fiji-ImageJ). Different letters indicate statistically significant differences between samples in a One-way ANOVA, with Tukey's multiple comparison test, $P < 0.05$.

Table S1. Description of interacting proteins of SlDRPs.

Sol ID	Description	Interpro domain(s)	Interaction Score			
			SlDRP1A	SlDRP1B	SlDRP1C	SlDRP2A
Solyc05g023750	Arf-GAP with GTPase, ANK repeat and PH domain-containing protein 3 (AHRD V1 *--* AGAP3_HUMAN)	IPR001164 Arf GTPase activating protein	3,1979	3,1354	-	-
Solyc07g047770	Histidine kinase 1 (AHRD V1 ***- D9ZHC0_HYPPE)	IPR006189 CHASE	2,3125	2,0000		
Solyc06g053760	Syntaxin (AHRD V1 ***- Q6X9V9_HORVD)	IPR010989 t-SNARE	2,3021	2,0104	-	-
Solyc03g119010	Pre-mRNA-splicing factor SPF27 (AHRD V1 **-- D3PJ34_9MAXI)	IPR008409 Breast carcinoma amplified sequence 2	1,8542	2,1458	-	-
Solyc02g069680	Charged multivesicular body protein 2a (AHRD V1 ***- B6TQT7_MAIZE)	IPR005024 Snf7	1,8542	1,5938	1,7813	1,6354
Solyc09g014450	Unknown Protein (AHRD V1)	IPR000996 Clathrin light chain	1,5625	1,5833	3,6042	1,3229
Solyc10g078790	At5G57460 protein (Fragment, TPLATE complex subunit involved in clathirin mediated endocytosis). (AHRD V1 *-*- B9DH18_ARATH)		-	-	3,1458	-
Solyc01g108800	3-hydroxyisobutyryl-CoA hydrolase (AHRD V1 **** D3NUG5_AZOS1)	IPR001753 Crotonase, core	-	-	2,3750	-
Solyc05g010460	Unknown Protein (AHRD V1)	IPR010683 Protein of unknown function DUF1262	-	-	2,3646	-
Solyc05g010470	Unknown Protein (AHRD V1)	IPR010683 Protein of unknown function DUF1262	-	-	2,3646	-
Solyc01g080930	Enoyl-CoA-hydratase (AHRD V1 ***- Q9SE41_AVIMR)	IPR001753 Crotonase, core	-	-	2,1354	-
Solyc05g026050	Charged multivesicular body protein 4b (AHRD V1 ***- B6UES0_MAIZE)	IPR005024 Snf7	-	-	2,0625	-

Table S2. Interactome interaction scores.

PTIR AC	Protein A		Protein B		Score
	Sol ID	Protein Name	Sol ID	Protein Name	
PTIR258763	Solyc06g053760	K4C663	Solyc06g053760	K4C663	9,9063
PTIR230541	Solyc05g023750	K4BZL4	Solyc05g023750	K4BZL4	9,4271
PTIR293829	Solyc07g047770	K4CEY3	Solyc07g047770	K4CEY3	7,9375
PTIR230920	Solyc05g026050	K4BZZ2	Solyc05g026050	K4BZZ2	7,4063
PTIR078557	Solyc01g108800	K4B395	Solyc01g108800	K4B395	7,0000
PTIR042343	Solyc01g080930	K4AXY5	Solyc01g080930	K4AXY5	6,9063
PTIR042347	Solyc01g080930	K4AXY5	Solyc01g108800	K4B395	6,6667
PTIR322002	Solyc09g014450	K4CRS2	Solyc09g014450	K4CRS2	6,4271
PTIR055395	Solyc01g095970	K4AZY6	Solyc01g095970	K4AZY6	5,4063
PTIR233141	Solyc05g050600	K4C192	Solyc05g050600	K4C192	5,4063
PTIR233145	Solyc05g050600	K4C192	Solyc11g039650	K4D7U2	5,0417
PTIR055399	Solyc01g095970	K4AZY6	Solyc05g050600	K4C192	4,9479
PTIR055403	Solyc01g095970	K4AZY6	Solyc11g039650	K4D7U2	4,7292
PTIR019493	Solyc01g005310	K4AS98	Solyc11g039650	K4D7U2	4,6250
PTIR102183	Solyc02g069680	K4B7V0	Solyc02g069680	K4B7V0	3,8646
PTIR019491	Solyc01g005310	K4AS98	Solyc09g014450	K4CRS2	3,6042
PTIR102192	Solyc02g069680	K4B7V0	Solyc05g026050	K4BZZ2	3,5313
PTIR055398	Solyc01g095970	K4AZY6	Solyc05g023750	K4BZL4	3,1979
PTIR019492	Solyc01g005310	K4AS98	Solyc10g078790	K4D2C0	3,1458
PTIR230542	Solyc05g023750	K4BZL4	Solyc05g050600	K4C192	3,1354
PTIR322003	Solyc09g014450	K4CRS2	Solyc10g078790	K4D2C0	2,4896
PTIR019486	Solyc01g005310	K4AS98	Solyc01g108800	K4B395	2,3750
PTIR019488	Solyc01g005310	K4AS98	Solyc05g010460	K4BXL9	2,3646
PTIR019489	Solyc01g005310	K4AS98	Solyc05g010470	K4BXM0	2,3646
PTIR055401	Solyc01g095970	K4AZY6	Solyc07g047770	K4CEY3	2,3125
PTIR055400	Solyc01g095970	K4AZY6	Solyc06g053760	K4C663	2,3021
PTIR182495	Solyc03g119010	K4BM50	Solyc05g050600	K4C192	2,1458
PTIR019485	Solyc01g005310	K4AS98	Solyc01g080930	K4AXY5	2,1354
PTIR019490	Solyc01g005310	K4AS98	Solyc05g026050	K4BZZ2	2,0625
PTIR233142	Solyc05g050600	K4C192	Solyc06g053760	K4C663	2,0104
PTIR233143	Solyc05g050600	K4C192	Solyc07g047770	K4CEY3	2,0000
PTIR055396	Solyc01g095970	K4AZY6	Solyc02g069680	K4B7V0	1,8542
PTIR055397	Solyc01g095970	K4AZY6	Solyc03g119010	K4BM50	1,8542
PTIR019487	Solyc01g005310	K4AS98	Solyc02g069680	K4B7V0	1,7813
PTIR102211	Solyc02g069680	K4B7V0	Solyc11g039650	K4D7U2	1,6354
PTIR102193	Solyc02g069680	K4B7V0	Solyc05g050600	K4C192	1,5938
PTIR233144	Solyc05g050600	K4C192	Solyc09g014450	K4CRS2	1,5833
PTIR055402	Solyc01g095970	K4AZY6	Solyc09g014450	K4CRS2	1,5625
PTIR322004	Solyc09g014450	K4CRS2	Solyc11g039650	K4D7U2	1,3229

Table S3. Co-expressed genes list of SlDRP1B, SlDRP1C, SlDRP2A. (Attached xls)

Table S3 available in a separate excel file.

Table S4. Gene ontology analysis.

PANTHER GO Overrepresentation Test among 180 mapped genes from 191 SIDRP1B co-expressed genes. Table shows the first ten GO term with lowest p-Value, represented in bubble chart in Figure 1D.

GO biological process complete	Count in gene set	Fold enrichment	P-value
vesicle-mediated transport (GO:0016192)	25	12.28	2.18E-19
localization (GO:0051179)	37	5.36	9.29E-17
transport (GO:0006810)	33	5.48	3.05E-15
establishment of localization (GO:0051234)	33	5.35	5.89E-15
protein metabolic process (GO:0019538)	38	4.43	1.29E-14
cellular protein metabolic process (GO:0044267)	37	4.54	1.53E-14
organonitrogen compound metabolic process (GO:1901564)	43	3.50	4.69E-13
cellular localization (GO:0051641)	24	6.68	4.75E-13
cellular macromolecule metabolic process (GO:0044260)	41	3.63	6.05E-13
macromolecule metabolic process (GO:0043170)	43	2.86	3.11E-10

Table S5. List of specific real-time PCR primers used for SlDRP1s gene expression analysis.

Name	Target	Sequence
qSlDRP1A_F	Solyc01g095970	GGGCTAAGTAGACACAAACC
qSlDRP1A_R	Solyc01g095970	AGAATGACACCTATCCCTACA
qSlDRP1B_F	Solyc05g050600	GATTCAGTAGCATGGTCTAAGTAA
qSlDRP1B_R	Solyc05g050600	AGAATCACTGGTCACCTATCT
qSlDRP1C_F	Solyc01g005310	GCAGTTGCTTGGAAATGATG
qSlDRP1C_R	Solyc01g005310	CGATCACTCTACTCTACTCGAT
qSlDRP1D_F	Solyc08g077360	CCTCATATGTTGGGATGGTG
qSlDRP1D_R	Solyc08g077360	TTTGCCCAGTTGCGTATAG
